# Improving transparency in malaria programme funds: A business case for connected diagnostics in Kenya

**DOI:** 10.4102/jphia.v15i1.653

**Published:** 2024-11-29

**Authors:** Charlotte M. Dieteren, Emmanuel Milimo, Angela Siteyi, Shannen van Duijn, Leon Stijvers, Lilyana Dayo, Gregory Ganda, Tobias F. Rinke de Wit

**Affiliations:** 1 PharmAccess Foundation, Amsterdam, the Netherlands; 2 Amsterdam Institute for Global Health and Development (AIGHD), Amsterdam, the Netherlands; 3 PharmAccess Foundation, Nairobi, Kenya; 4 Kisumu County Department of Health, Kisumu, Kenya

**Keywords:** ConnDx, health financing, vertical funds, efficiency, digitalisation

## Abstract

**Background:**

International vertical health financing programmes risk functioning in parallel with existing domestic funding in low- and middle-income countries (LMICs), leading to inefficient service delivery and concomitant poor health outcomes.

**Aim:**

We assessed the opportunities offered by digitalised diagnostics (ConnDx) to target and monitor health funds to those in need objectively and transparently.

**Setting:**

ConnDx was rolled out in five private health facilities in Kisumu, Kenya.

**Methods:**

The ConnDx process was codeveloped with the local Department of Health of Kisumu. We used the quantitative data generated by ConnDx. We also calculated the costs for ConnDx and standard care to assess potential cost reductions.

**Results:**

In total, 2199 malaria cases were detected among 11 689 patients with fever. ConnDx allowed for the identification of malaria hotspots, semi-real-time assessment of patient health seeking behaviour across facilities and insights in doctor’s prescription behaviours. Based on these insights, we estimated a 25% reduction in costs can be realised, while simultaneously better quality indicators can be monitored.

**Conclusion:**

The concept of ConnDx can be used for any medical condition that can be diagnosed in a digital manner and linked to mobile payment systems. The generated data can contribute to better quality services for individual patients while at the same time support local health policy makers and managers for more targeted interventions.

**Contribution:**

The ConnDx approach can help decision makers in LMICs to channel disease-specific funds to the right patients for the right disease at the right time, which can potentially accelerate the way to universal health coverage.

## Background

Universal Health Coverage (UHC) is set as one of the targets of the 2030 Sustainable Development Goals (SDGs), adopted by all United Nations (UN) members in 2015. Universal Health Coverage means that everybody can access quality health services when in need and without financial hardship.^1^ Many low- and middle-income countries (LMICs) experience major challenges in the UHC journey, struggling to provide even a minimum basic package of care because of a lack of sufficient funding. Risk pooling and prepayment for health (health insurance) are key instruments to achieve UHC as they provide financial protection against individual catastrophic healthcare expenditures. Insurance can be successful only when quality healthcare is available to guarantee coverage of essential health services, as this supports clients’ trust and willingness to prepay. Although domestic health insurance is on the rise in LMICs, other health financing mechanisms remain in place. For example, in Kenya, out-of-pocket payments and external funding remain dominant financial resources.^2^ The latter, external funding, tends to be fragmented, donor- or disease-specific.^2^ While these so-called ‘vertical’ programmes are usually highly specific with defined objectives and therefore easier to evaluate, they also often function in parallel and thus contribute to fragmented health systems.^3^ Such deficiencies result in poor health outcomes,^4^ and call for increased efficiencies in healthcare financing.

Diagnostics only covers 5% – 10% of all healthcare costs but leads to 60% – 70% of all healthcare decisions.^5^ Hence, creating more efficiency in the diagnosis stage of vertical disease programmes could potentially lead to more efficient targeting of disease-specific funds. Accurate disease diagnostics are an inevitable facet of a good functioning continuum of care, as it usually serves as the starting point of a treatment trajectory. Diagnostics is not only crucial on an individual level but also on a societal level because monitoring and early detection of infectious diseases is required to control a potential outbreak.

The global digital revolution has the potential to play a significant role in reducing the current deficiencies in vertical disease-specific funds. The Internet of Things (IoT) is developing rapidly and includes tools and devices that can measure human indicators and upload to cloud-based databases.^6^ This also applies to diagnostic tools, as these become increasingly more digital and involve the usage of artificial intelligence (AI). For example, a smartphone application can screen self-taken images of the cervix of cancer,^7^ or distinguish a cough as an upper or lower respiratory tract infection based on the sound.^8^ Mobile phones make diagnostics cheaper, more connected, more patient-oriented, while maintaining good levels of sensitivity and specificity and adding options for independent (AI-assisted) external quality control.^9^ The portable nature of these technologies also enhances patient access by minimising travel time.^10^ The promise of digital diagnostics is huge and can lead to disruptive changes in the health system landscape in LMICs, while adequate scientific evidence of its effects remains essential.^11^ The evidence in high-income settings is growing,^12^ and also in sub-Saharan Africa (SSA) the importance is recognised and different forms of mHealth and digital diagnostics have shown to play a major role in improving effective delivery and management of both non-communicable diseases (NCDs) and communicable diseases (CDs).^13,14,15,16^

In LMICs, digital connected diagnostics (ConnDx) have an ever-increasing potential as for the first time in 2022 half of the population is using mobile Internet and this number is still growing.^17^ Mobile health (mHealth) covers a broader spectrum besides diagnosis; studies in SSA have demonstrated that mHealth is also beneficial in stimulating health workers performance, treatment adherence and educational purposes.^18^ The emergence of digital payment mechanisms – called ‘bankless banking’ – completes the circle of digital diagnostics as it allows for a complete digital medical consultation. Mobile payments for goods and services can be made without using cash, cheques or cards. One example of a mobile money transfer service is M-PESA (mobile money; PESA means money in Swahili), which was first launched in 2007 in Kenya and today has more than 51 million customers and processed monthly 614m transactions in Kenya, Tanzania, Mozambique, Democratic Republic of the Congo (DRC), Lesotho, Ghana and Egypt.^19,20^

The ingredients of the above discussed *global digital revolution –* IoT, digitalisation of diagnostics, revolutionary mobile phone penetration and mobile bankless banking – contributed to the development of our concept ‘Connected Diagnostics’ (ConnDx).^21^ ConnDx uses digital diagnostics to predict or diagnose a medical condition and channel funds for monitoring, treating or preventing that same condition to individual patients and clinics.^21,22^ During this process, semi-real-time data are collected both from patients and healthcare providers allowing for increased efficiencies and transparency through presentation on dashboards for health policymakers. The feasibility, user experience and clinical performance of ConnDx in Kenya have been documented elsewhere.^22^ Africa can leverage innovations in diagnostics, data connectivity and mobile technology to enhance disease surveillance, control and outbreak response and therefore ConnDx is considered as the future of disease control in Africa^23^ In this article, we aim to assess the opportunities offered by ConnDx to target and monitor health funds to those in need objectively and transparently. In this study, we use malaria as a showcase, but more general application is possible for medical conditions in LMICs that are (at least partly) financed by (inter)national disease-specific funds.

## Research methods and design

This study builds upon the experiences of an implemented ConnDx approach in Kenya.^22^ We use retrospective quantitative data collected in the ConnDx field experiment to demonstrate how a ConnDx approach can contribute to channel financial health funds.

### Study setting

The ConnDx field experiment was conducted in Kisumu, Western Kenya, which is one of the counties with the highest malaria prevalence of the country. Although recent figures point towards a prevalence decrease from 27% in 2015 to 19% in 2021, these figures remain substantial and could worsen again with ongoing climate-change.^24,25^ Malaria misdiagnosis in Kisumu was previously determined to be very high, at 47%.^26^ Currently, Kisumu is rolling out a social health insurance programme (Marwa) with most of its population (~1m) subscribed with CarePay M-TIBA (mobile health wallet; TIBA means care in Swahili) through their mobile phones with a high penetration rate.^27^

### Study period and study sample

The experiment ran from October 2017 to December 2018 across five health facilities, where ConnDx was presented as a ‘Malaria Test and Treat Campaign’ to improve access to quality malaria diagnosis and treatment. The technical evaluation of this experiment is reported elsewhere.^22^ The selection of providers was determined by factors such as high patient throughput recorded in District Health Information Software-2 (DHIS)-2 proximity to water in their geographic location and the willingness of the management to participate. The included providers varied from smaller facilities with specific operating hours to large 24/7 hospital facilities. Patients with malaria symptoms were informed of the campaign through posters in the waiting rooms and verbally during consultations with clinicians. The experiment operated alongside existing systems.

### The ConnDx process for malaria in Kenya

Pilot work on ConnDx was performed both in Samburu County (for malaria and brucellosis)^28^ and later in Kisumu County (for malaria).^22^ In Samburu we tested the usage by community health workers of a digital ‘fever gun’ to identify febrile patients who would quality for our ConnDx rapid blood tests. This approach proved not only successful but also appeared too labour intensive and not scalable. In Kisumu, we subsequently tested a digital linkage between the results of a rapid diagnostic test (RDT) digitalising device (Deki Reader) and a digital mobile health wallet (M-TIBA) to target payments for malaria treatment. This proved successful and inspired this study aiming at a potential business model for scaling.

**FIGURE 1:**
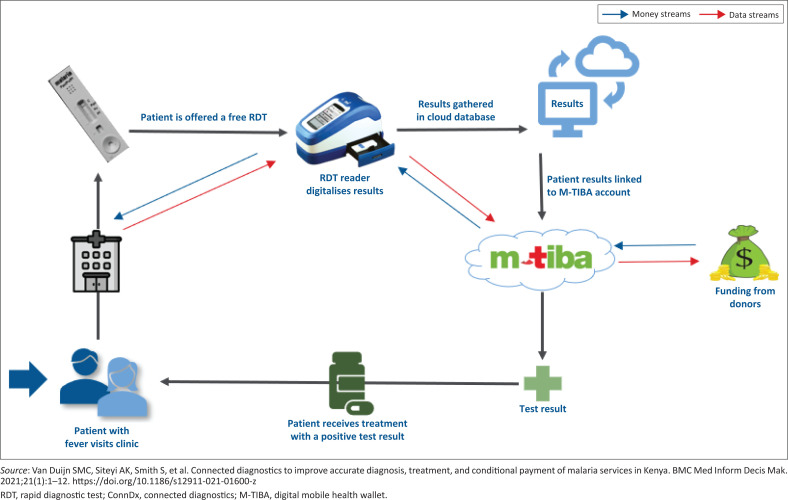
Visualisation of the ConnDx process for malaria.

The M-PESA – the mobile money transfer system in Kenya – laid the foundations for M-TIBA: a healthcare exchange platform that collects real-time data on healthcare transactions creating transparency and de-siloes information from providers, patients and payers (www.mtiba.com). For patients, M-TIBA serves as a mobile health wallet. In countries where most pay for care out-of-pocket, M-TIBA provides options for bundling different payments sources into prepayment, create risk pools and linkage to insurance that can have a big impact on health seeking behaviour. We have applied the ConnDx process for malaria,^22^ but ConnDx could be applied to any medical condition that can be digitally diagnosed and for which vertical funding exists. Figure 1 visualises the ideal ConnDx process for malaria. In our first pilots many of these steps were put into practice, as described next.

The process starts when a patient presents with fever at a health facility and the clinician suspects malaria. The patient can be invited to enrol into M-TIBA using his or her personal phone, which enables digital collection of clinical information and transactions into a cloud database. Subsequently, the clinician refers the patient to the lab for a malaria RDT. The lab technician performs the RDT and can interpret the result in real-time and inform the clinician to take pertinent action. In addition, the lab technician inserts the RDT into a network-connected reader that makes a digital photograph in a standardised manner and uploads the data into a dedicated cloud database (we used a reader from FioNet, Figure 1). Alternatively, the lab technician uses a smartphone application (App) to photograph the RDT result and upload accordingly, while AI software interprets the picture. The reader/App should feature acceptable sensitivity and specificity, be able to operate without electricity for a considerable time,^29^ allow for off-line data collection and can be electronically maintained from a distance and be validated in LMIC settings.^30,31,32^ In the next step, the RDT cloud database exports its information to a payment platform using customised application programming interfaces (APIs). An anonymised unique identifier-code is used (e.g., M-TIBA transaction code) that enables the combination of data from both platforms according to General Data Protection Regulation (GDPR) regulations. The combined data allow for downstream conditional payment algorithms: funds to the providers for performing RDTs (on the condition of correct performance) and funds for the patients for first or second line malaria treatment (on the condition of being tested malaria positive and according to the applicable National Guidelines). Simultaneously, important data are collected and fed back into the system, like numbers, timing and geographical location of positive malaria cases, timing of provision of medical services, patient behaviour in terms of selecting providers, provider behaviour in terms of prescription of antimalarials (generic/branded, first line, second line).

### Data analyses – Variables of interest

#### Identification of hotspots and vulnerable groups

To assess the opportunities of ConnDx to contribute to improved intervention targeting we wished to assess its ability to identify hotspots and vulnerable population groups. We used the timing and geographical location of positive malaria tests to identify hotspots. For the identification of vulnerable groups, we estimated the socio-economic status (SES) by using three indicators (access to electricity, toilet type and education level of household head). These SES indicators were previously identified as most informative, based on their strong association with the wealth index computed through principal component analysis in the Multiple Indicator Cluster Surveys (MICS) dataset, which classified households into three wealth categories.^24^ Further details on this measurement are described elsewhere.^22^

#### Identification of patient healthcare seeking behaviour

The ConnDx field experiment generated insights in patient’s travel distances to clinics because it registered geographic coordinates of participating clinics as well as the community units where patients live. Accordingly, we estimated which facilities were chosen by the patients and what the average distances travelled from their home was. The actual location of homes was not known; however, the participants indicated to which Community Unit they belonged (aggregate group of ~5000 citizens). The geo-position of their homes was assumed to be in the geographical middle of the pertinent Community Unit they are living in. In addition, we also reported the time and day of the diagnostic performance, which we analysed to identify peak hours.

#### Measuring health staff compliance to guidelines

The ConnDx field experiment provided data on prescription behaviour by providers. Prescription of antimalarial drugs is recorded in the Kenyan National Malaria guidelines and should follow strict procedures regarding body weight of the patient and choice of first line versus second line medicines, usually in the 95%/5% range.^33^ Discrepancy from these guidelines was quantified.

#### Estimation cost model

We created a cost model to generate insights into the standard care and ConnDx care approach. For this model we used the data insights regarding unnecessary overprescription of antimalarial. Additionally, we made some reasonable assumptions in the model based on the findings of the ConnDx pilot. We assumed ConnDx would support lowering the usage of microscopy while increasing the RDT usage (ratio change of 50:50 to 20:80) because of RDT’s user-friendliness, shorter time-to-result, less dependence on qualified laboratory staff, electricity, equipment and reagents. Costs of uploading data into the digital RDT reader and eventually into a digital payment platform such as M-TIBA are both set at a reasonable 10% of diagnostics costs. We assumed that with active promotion of ConnDx the annual malaria population testing rate in Kisumu would (slightly) increase from the current 40% – 50%. For current annual antimalarials usage rate the figure of 46% was used (based on paediatric data^24^). Under ConnDx ideally only those who test positive will receive treatment, thus usage decreasing to the currently reported decreased malaria prevalence of 19%. During our field experiment we observed in participating private facilities the ratio first/second line antimalarial drugs to be 75%/25%, respectively. We assumed that with implementation of ConnDx this ratio could change to 95% and 5% because of improved adherence to treatment National Guidelines. We also assumed that sensitivity and specificity of microscopy and RDT are comparable in LMIC field situations,^25^ although microscopy remains the gold standard. The analyses were carried out in Microsoft Excel and the visuals were generated in powerBI.

### Ethical considerations

Ethical approval to conduct this study was obtained from the Directorate of the Department of Health in Kisumu on July 19, 2018. This study describes an operational initiative implemented in Kisumu, Kenya, in close collaboration with the local Department of Health. This initiative was not structured as a formal research study and thus formal ethical clearance was waived. Approval for this project was granted by the County Chief Office of Health. Participants in the programme were verbally informed by their healthcare provider. By opting to participate, participants enrolled in the M-TIBA digital mobile health platform, which includes an informed consent question during initial registration. To safeguard the confidentiality of both research subjects and personnel records, data collection was anonymised. This article does not disclose any individual participant data in any format.

## Results

During the experiment period (2017–2018), ConnDx detected 2119 positive malaria cases among a sample of 11 689 patients with fever who reported for routine fever services and subsequently were tested using RDTs. This means that we report a facility-detected malaria prevalence of 18%. The background characteristics of the sample are reported elsewhere.^22^ Further in the text we report on the four identified areas within ConnDx that could contribute to objectively target and monitor health funds to those in need. Findings have the potential to be extrapolated to other medical conditions and settings in LMICs.

### Identification of hotspots and vulnerable groups

**FIGURE 2:**
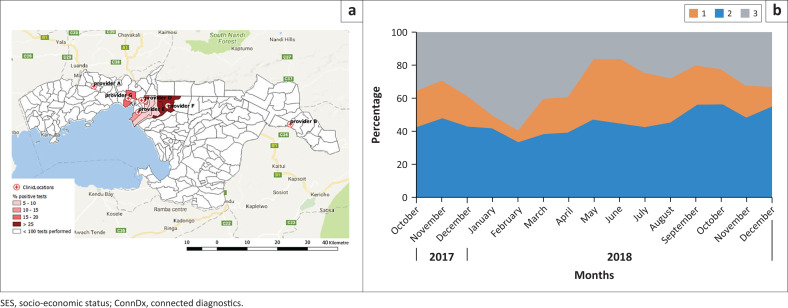
(a) Hotspots of malaria in Kisumu County (% positive tests); (b) Socio-economic status of ConnDx participants (level 1 = lowest SES).

The field experiment allowed for the timely identification of malaria hotspots (Figure 2a). It monitored rainy season fluctuations in malaria incidence, and it helped providing a first indication from which socio-economic groups of the population benefitted (Figure 2b). Those with the lowest SES had increased ConnDx usage over time, possibly because of increasing awareness of its (free) services. Overall, 1132 out of 2119 (51%) malaria positive cases were identified among those with the lowest SES (Figure 2b).

### Insights on patient healthcare seeking behaviour

**FIGURE 3:**
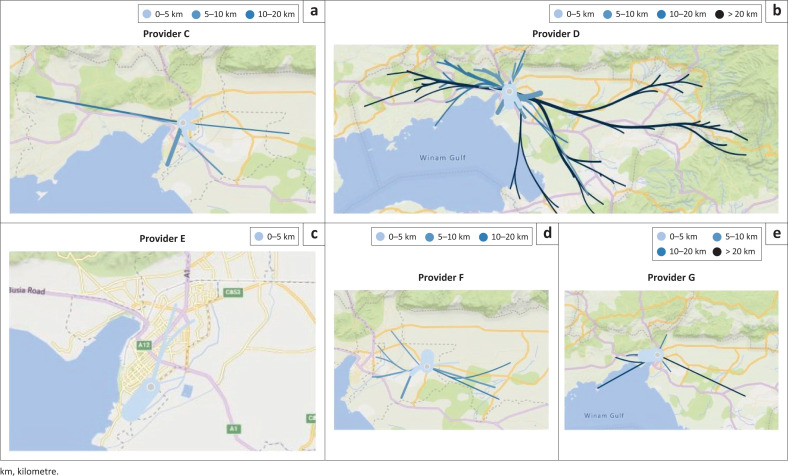
Patient travel distances to the healthcare provider.

**FIGURE 4:**
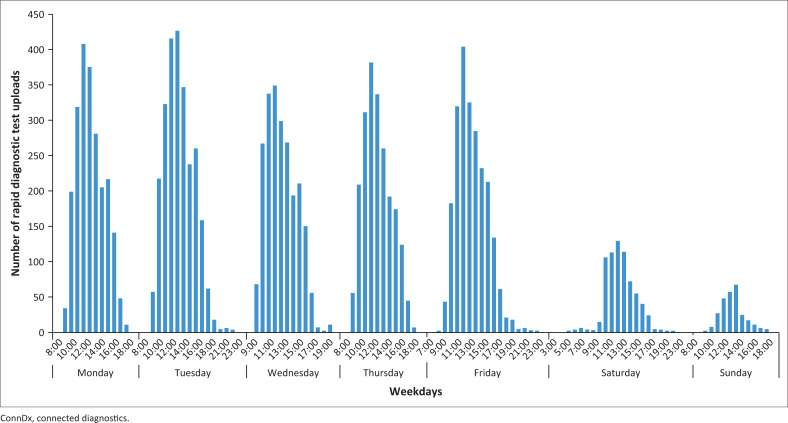
Provider rush hours (in ConnDx rapid diagnostic test uploads per hour and per day).

The geographic coordinates of participating clinics as well as the community units where patients’ live were used to gain insights in patients travel distance to the clinic. Facilities were chosen by patients and recorded in real time and the average distances travelled from their home could be calculated. Figure 3 shows the patient travel behaviour profiles for each provider. The thickness of the blue lines indicates the number of patients. For each provider the thickest line is light blue, demonstrating that the majority of the sample had to travel less than 5 km for the facility visit. Providers G and D receive patients with a wide variety in travel distances, including patients who had to travel for more than 20 km. Figure 4 depicts the time of the day of RDT performance and data uploading, which can serve as a proxy for laboratory peak hours. Clearly weekdays, particularly Mondays and Tuesdays, were the busiest days and 10:00–14:00 the busiest times.

### Health staff compliance to guidelines

**FIGURE 5:**
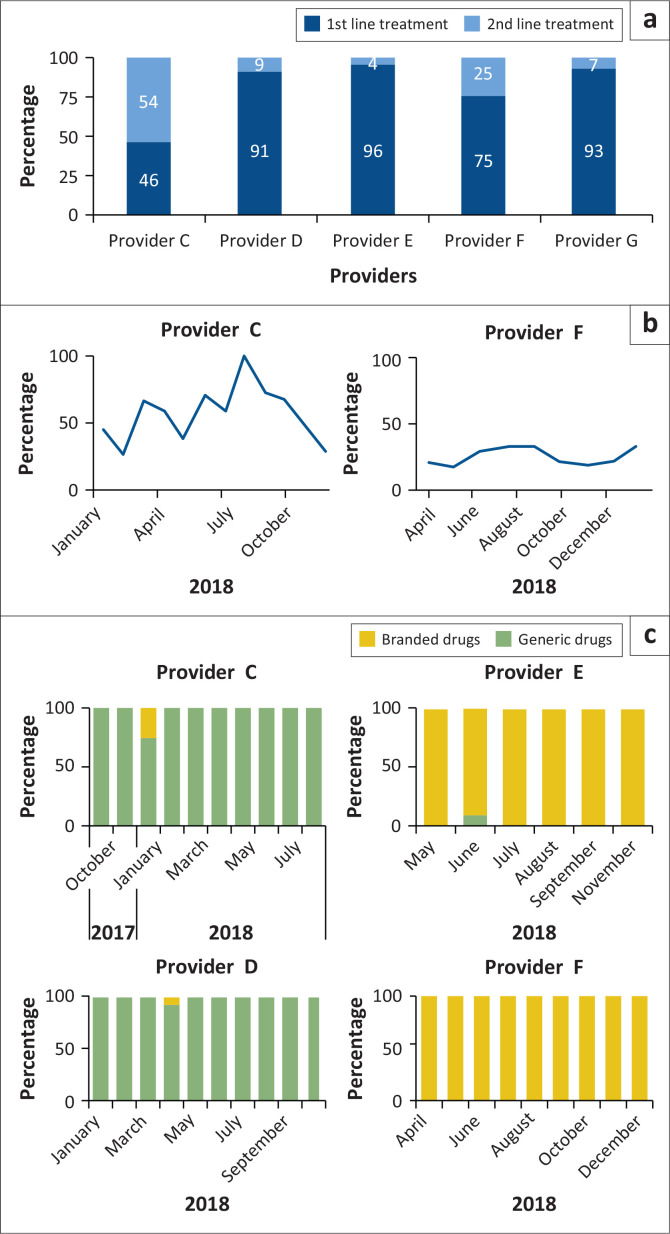
(a) Percentage first line versus second line malaria treatment prescriptions per provider; (b) time-dynamics of second line malaria treatment prescriptions for Provider C and Provider F; (c) usage of generic versus branded malaria drugs per Provider (C, E, D and F).

Figure 5a depicts substantial differences between first line and second line medicines of the five providers that remained active during the entire project. Two providers dropped out after 2 months because their experienced workload was too much. The figure shows unusually high frequencies of second line medicines prescribed by Provider C (54%) and Provider F (25%). Figure 5b demonstrates the time-dependency of this second line prescription, with relatively consistent 25% second line prescription practice by Provider F and peaks of even 100% for Provider C. Figure 5c depicts the choices of branded versus generic malaria drugs, apparently dichotomous, with Providers C and D almost exclusively prescribing generic drugs and Providers E and F using mostly branded antimalarials.

### Cost estimation model: Standard – ConnDx

Based on the data and insights discussed in the previous paragraphs, we were able to formulate a preliminary cost reduction model applicable to Kisumu County that considers malaria commodities and focuses on the potential to reduce unnecessary overprescription of antimalarials, while increasing diagnostic services (Table 1). Considering the assumptions listed in the method section, we predict a 25% cost reduction for Kisumu County when ConnDx would be standardly used under the guidance of the Department of Health. In our view this model represents a bare minimum.

**TABLE 1:** Cost comparison model of *ConnDx* for Kisumu County based on data gathered during the pilot in Kisumu in 2018.†

Model characteristics	Population characteristics	Standard	ConnDx	Price ($)	Costs Δ	Standard ($)	ConnDx ($)
**Population Kisumu**							
Total population‡	1 155 574	-	-	-	-	-	-
Children/individuals (< 35 kg)	25%	-	-	-	-	-	-
Malaria prevalence Kisumu§	19%	-	-	-	-	-	-
**Behaviours**							
Annual malaria Dx testing rate	-	40%	50%	-	-	-	-
Annual malaria Rx usage rate††	-	46%	19%	-	-	-	-
Drug prescription choice for first line	-	75%	95%	-	-	-	-
Drug prescription choice for second line	-	25%	5%	-	-	-	-
**Prices Dx**		-	-				
Microscopy test	-	50%	20%	0.78	-	-	-
RDT	-	50%	80%	1.17	-	-	-
Top-up Dx reader (10%) + M-TIBA (10%)	-	0%	80%	0.23	-	-	-
Average Dx price	-	0.98	1.28	-	-	-	-
**Prices Rx¶**							
Antimalarials (first line kids)	-	-	-	0.59	-	-	-
Antimalarials (second line kids)	-	-	-	2.34	-	-	-
Average Rx kids	-	**1.02**	**0.67**	-	-	-	-
Antimalarials (first line adults)	-	-	-	1.17	-	-	-
Antimalarials (second line adults)	-	-	-	3.90	-	-	-
Average Rx adults	-	1.85	1.31	-	-	-	-
**Extrapolation to population Kisumu**							
Dx	-	-	-	-	64%	450 674	739 105
Rx children	-	-	-	-	−73%	136 047	36 927
Rx adults	-	-	-	-	−71%	738 542	215 140

**Total**	**-**	**-**	**-**	**-**	**-25%**	**1 325 263**	**991 173**

Note: Please see the full reference list of this article for more information. The bold data in Prices Rx reflect the outcome of the related prices × the figures in the behaviors section. The figures in the total row: -25% is average risk reduction, reflected in the bold figures next to it, if the input of the model was applied to the population of Kisumu.

RDT, rapid diagnostic test; ConnDx, connected diagnostics.

† , Exchange rate Kenyan shilling to United States dollar ($) was 0.0078 (6 March 2023);

‡ , https://kenya.hurumap.org/profiles/county-42-kisumu/^34^;

§ , World Health Organization, 2022.^35^;

¶ , Based on M-TIBA observations;

†† , Otambo et al., 2022.^36^

## Discussion

In this study we utilised retrospective quantitative data from a ConnDx project^22^ to evaluate the opportunities provided by digitalised diagnostics in targeting and monitoring health funds objectively and transparently for those in need. We identified four areas within ConnDx that could contribute to more efficiency in vertical malaria funds: (1) the identification of hotspots and vulnerable groups; (2) insights in patients’ healthcare seeking behaviour; (3) insights in provider prescription behaviour and (4) the potential to significantly reduce costs. Findings have the potential to be extrapolated to other medical conditions and settings in LMICs.

The following implications are drawn from the four key opportunities identified through the use of digitalised diagnostics. The identification of hotspots and vulnerable groups are in particular useful to target malaria interventions, like distribution of bed nets and timely identification of risk populations and geographic areas with needs for antimalarials. Targeting is important when resources of health authorities are limited, so they can make best-possible informed decisions, where and to whom malaria supplies should be made available. Information regarding which facilities patients choose to visit is valuable for healthcare providers to better manage patients, for example by directing patients to less-occupied facilities via SMS messaging, deploying more staff during rush hours, thus reducing the burden on the already highly occupied staff and reducing waiting times for patients. In addition, ConnDx can monitor health staff compliance to guidelines and lab standard operating procedures (SOPs) in semi-real-time. This allows managers to target quality interventions such as training of lab technicians or clinicians or improvements in drug supply chain management. For policy makers, this information provides important insights in actual compliance with malaria guidelines. Finally, while we already show a potential cost reduction of 25% there are other saving opportunities that we did not enumerate in this study. For example, through the increased digitalisation (decreased paperwork) feature of ConnDx, less dependence on expensive equipment (microscopes), their maintenance and electricity consumption and more opportunities for lower trained and less salaried laboratory staff to provide diagnostic services.

The concept of ConnDx can in principle be used for any medical condition that can be diagnosed in a digital manner. This could be cataract, cervical cancer or skin cancer that can be diagnosed through mobile phones photography.^7^ It could also be applied to NCDs through digital blood sugar and hypertension measurements.^37^ Moreover, ConnDx can be applied to all RDTs that are (being) developed for blood, plasma, swabs, urine samples through photographic digitalisation using mobile RDT Reader Apps.^38^ We recommend developing ConnDx approaches for medical conditions that are highly prevalent or very debilitating in LMICs, that are treatable with available and affordable medicines, preferably in a point-of-care setting. However, scaling ConnDx to other diseases may require significant adaptations in the platform’s design, clinical workflows and data capture mechanisms. As different diseases have different data needs, a careful design of ConnDx is required to accommodate these variations while maintaining efficiency.

There are several challenges in scaling the ConnDx approach across different settings. One of the primary challenges lies in infrastructure limitations, as reliable Internet access and the necessary technological infrastructure are crucial for the implementation. Implementing ConnDx at scale would also require coordination across multiple levels of government and health authorities to ensure that the system complies with local regulations. Addressing these challenges requires coordinated efforts from various stakeholders. A recent study in Uganda showed that it is possible to overcome these barriers by stakeholder engagement and promoting interoperability with the DHIS platform of the MoH.^39^ Moreover, it should be kept in mind that single testing for malaria entails the risk of overprescription of antibiotics, particularly in cases where patients have fever and test negative for malaria. This could contribute to accelerated antibiotic resistance and unnecessary additional costs.^40^

An efficiency leap can be achieved by mobile ConnDx when decision makers channel disease-specific funds to the right patients for the right disease at the right time. As value-based healthcare becomes more widespread, diagnostics will assume an increasingly crucial role, not only in terms of improved patient-centred quality of care but also in other aspects. ConnDx creates possibilities for credible, real-time outcome measures, both patient-reported and objectively diagnosed outcomes, which subsequently can be used to incentivise healthcare providers for following correct treatment trajectories and realising improved quality of patient services. Additionally, ConnDx offers the unique possibility of bottom-up funding for malaria service delivery, versus the current top-down mechanisms. This can be attractive to donors like the Global Fund, which are sometimes confronted with suboptimal usage of their resources in low resource settings.^41^ Using mobile phones of local providers close to the patient, malaria diagnostic data can be uploaded in semi-real-time, with geotagged global positioning system (GPS) coordinates, while money streams for appropriate malaria treatment can be monitored and accounted for. ConnDx thus potentially brings transparency in malaria diagnosis and treatment as well as in more targeted usage of funds to combat this disease. Donors such as President’s Malaria Initiative (PMI) and Global Fund to Fight AIDS, Tuberculosis and Malaria (GFATM) could adopt (an experiment where they put) malaria ConnDx as a conditional tool to recipients to qualify for prospective funds. Data sharing with stakeholders is facilitated by ConnDx through its feeding into digital dashboards using PowerBI that is freeware in its basic format. These dashboards can be easily installed in password-protected tablets and mobile phones of policy makers to allow them to make better-informed decisions on combatting malaria. Full transparency can be reached on weekly uploaded malaria test data by clinics, chemists, community health workers and pertinent remunerations allocated (data bundles, M-PESA funds, in kind RDTs). In addition, testing for malaria + other febrile diseases could further improve the ConnDx performance with respect to clinical decision making.^42^ Experiments along these lines are currently ongoing (working paper^43^).

When ConnDx becomes conditional for payment, it is at the same time an important means to pledge ‘vertical’ disease funding like PEPFAR, GFATM, PMI into ‘horizontal’ risk pools. Such risk pools are currently emerging in many countries in Africa (national social health insurances, health equity funds, health management organisations). Health insurance entities (such as National Health Insurance Fund in Kenya) could benefit from increased risk pools through pledges of vertical funds. Vice versa, these vertical funds could do this on the condition that certain pre-determined performance indicators are measured and reported in semi-real-time, which is exactly what ConnDx supports. This would bundle public and private funds into the same front-end prepayment pool, thus allowing for mutual enforcement (crowding in), while disease-specific outcome reporting to the disease-specific donor can be digitally realised at the backend. Vertical disease funds will continue to exist, because of human affinity with such topics, for example cancer funds, diabetes funds, rheumatoid arthritis funds. ConnDx can link these funds into general risk pools to achieve and finance UHC.

We acknowledge several limitations of this study. Firstly, the data in this study are from one Kenyan County and cover only five private providers (starting with seven and two of them phasing out after several months because of (perceived workload). This study was meant as a proof-of-principle and therefore participating providers were conveniently sampled and not representatively. Extrapolation of conclusions should be considered cautiously. On the other hand, private facilities provide almost half of all malaria services in Kenya with most of their data missing in the national Kenyan Health Information System resulting in a significant malaria information gap. For this reason, the current pilot was deemed important. Follow-up studies are currently performed to make ConnDx reader-free (using mobile phones as digitiser), broaden the suite of connectable malaria RDTs, extending the ConnDx approach to informal providers, like private chemists and community health works and remunerating correct usage of ConnDx with various (non-)monetary incentives. Secondly, the results of this study are based on real-world data which is on one hand a strength because it reflects reality, while on the other hand it is likely that our data are incomplete and filled in according to availability of provider’s (spare) time.

However, this holds true in general for provider reporting in clinical and diagnostic data to national databases (such as DHIS2), in particular private providers. Moreover, as the data only captured patients who visited the participating facilities, those febrile patients who reported in non-formal healthcare providers (chemists, drug shops) were missing. This represents an estimated 40% of malaria patients, as per Global Fund estimations.^44^ Therefore, the malaria positivity percentages, as obtained through this ConnDx approach, should not be interpreted as representative for local malaria prevalence data. Despite these limitations, the findings offer a meaningful reflection of real-world conditions, making them a robust foundation for identifying the opportunities presented by ConnDx. Thirdly, the routine malaria service comparison data used to estimate cost reductions are difficult to collect because of transparency uncertainties. This naturally influences the estimated cost reduction figure of 25%. However, for our calculations we applied the most conservative assumptions and for pricing the various elements of the model we used the most actual information we could collect. Finally, it should be stated that this was an observational study, not an intervention: ConnDx was performed in parallel to routine malaria service delivery; the results were not fed back to participating providers during roll-out. This implies that participating providers benefitted from extra income because of the parallel ConnDx malaria intervention they undertook. Interim feedback of ConnDx data analytics could have potentially further improved malaria service delivery. However, even in the absence of such feedback we already observed several improvements, for example. in reduced overprescription, likely because of Hawthorne effects.

## Conclusion

Several digital mobile revolutions in Africa contributed to the development of the concept of ConnDx. For the first time we were able to generate digital semi-real-time monitoring of personal health and healthcare data from private sector health facilities and use these to contribute to managing malaria, better target funds and at the same time collect intelligence that can improve quality of healthcare services that can lead to improved patient outcomes. This has the potential to fulfil a long-existing gap in national malaria information collection in African settings and linking private sector data to national systems, including DHIS-2. At the aggregate level ConnDx allows for the creation of dashboards that provide real-time (geographic) overviews of disease incidence, prevalence, socio-economic distributions and demographics of target populations to health managers and policy makers. Rapid decision making can thus be facilitated, like distribution of insecticide treated bed nets, malaria medicines and in the future vaccines. This enables a more efficient usage of otherwise scarce resources for healthcare in LMICs. ConnDx may accelerate our way to UHC by linking these scarce resources and funds into general risk pools.
